# Supplementary material for: Dual-wavelength metalens enables Epi-fluorescence detection from single molecules
Source: Nat Commun. 2024 Jan 2;15:26. doi: 10.1038/s41467-023-44407-4 (PMC10761847; doi:10.1038/s41467-023-44407-4)
Supplement: Supplementary file 1 — Supplementary Information [file 41467_2023_44407_MOESM1_ESM.pdf]

Supplementary Information

Dual-Wavelength Metalens Enables Epi-Fluorescence Detection from Single Molecules

Aleksandr Barulin,[1,2,#] Yeseul Kim,[3,#] Dong Kyo Oh,[3,#] Jaehyuck Jang,[4,5] Hyemi Park,[1,2] Junsuk Rho[3,4,5,6,]* and Inki Kim[1,2,]*

[1] Department of Biophysics, Institute of Quantum Biophysics, Sungkyunkwan University, Suwon 16419, Republic of Korea

[2] Department of Intelligent Precision Healthcare Convergence, Sungkyunkwan University, Suwon 16419, Republic of Korea

[3] Department of Mechanical Engineering, Pohang University of Science and Technology (POSTECH), Pohang 37673, Republic of Korea

[4] Department of Chemical Engineering, Pohang University of Science and Technology (POSTECH), Pohang 37673, Republic of Korea

[5] POSCO-POSTECH-RIST Convergence Research Center for Flat Optics and Metaphotonics, Pohang 37673, Republic of Korea

[6] National Institute of Nanomaterials Technology (NINT), Pohang 37673, Republic of Korea

*Corresponding authors: jsrho@postech.ac.kr; inki.kim@skku.edu

[#] These authors contributed equally to the work

Supplementary Note 1. Refractive index and extinction coefficient of meta-atom material

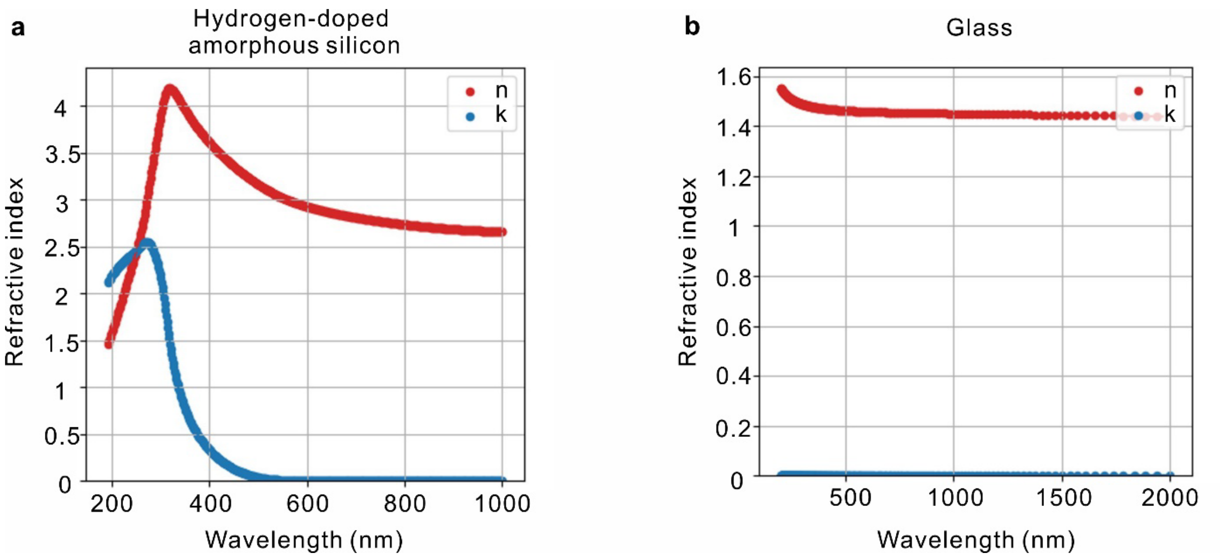

Supplementary Figure 1. Refractive index and extinction coefficient of (a) hydrogen-doped amorphous silicon and (b) glass (SiO_2). The refractive index data were measured using ellipsometry.

Supplementary Note 2. Theoretically ideal lens phase maps at 635 and 670 nm wavelengths

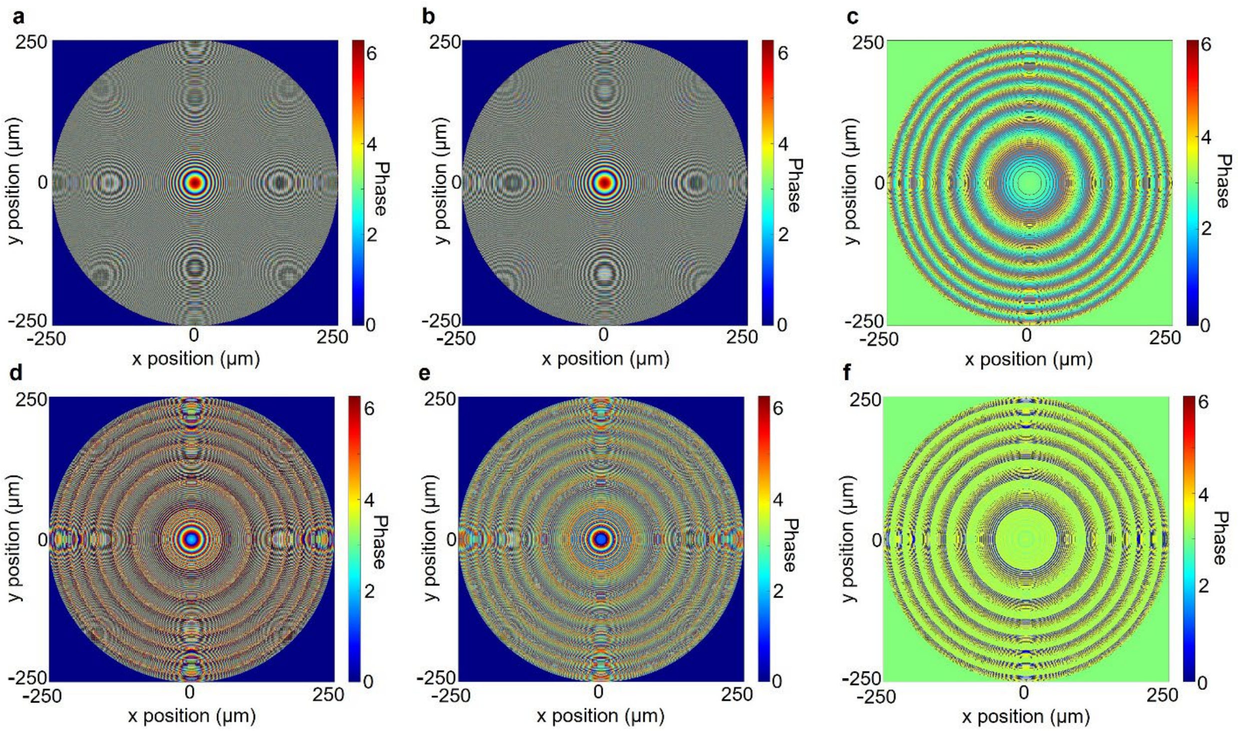

Supplementary Figure 2. (a), (b) Theoretically ideal phase map at 635 and 670 nm, respectively. (c) Phase difference between two ideal phase maps. (d), (e) Matched phase map at 635 and 670 nm, respectively. (f) Phase difference between two matched phase maps.

Supplementary Note 3. Principle of propagation phase

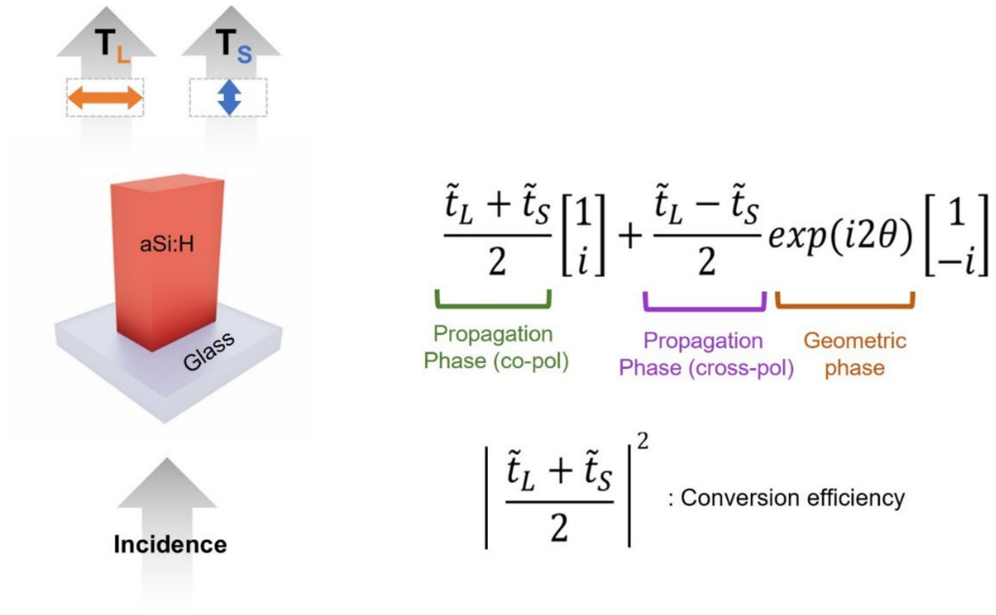

Supplementary Figure 3. Principle of propagation phase. The metalens modulates phase of incident light via co-polarization term. The phase variation between 0 and 2π is achieved owing to discrepancy in transmittance between the major and minor axes. The co-polarization term-based conversion efficiency exceeds that of cross-polarization term by around 5 %.

Supplementary Note 4. Meta-atom simulation results

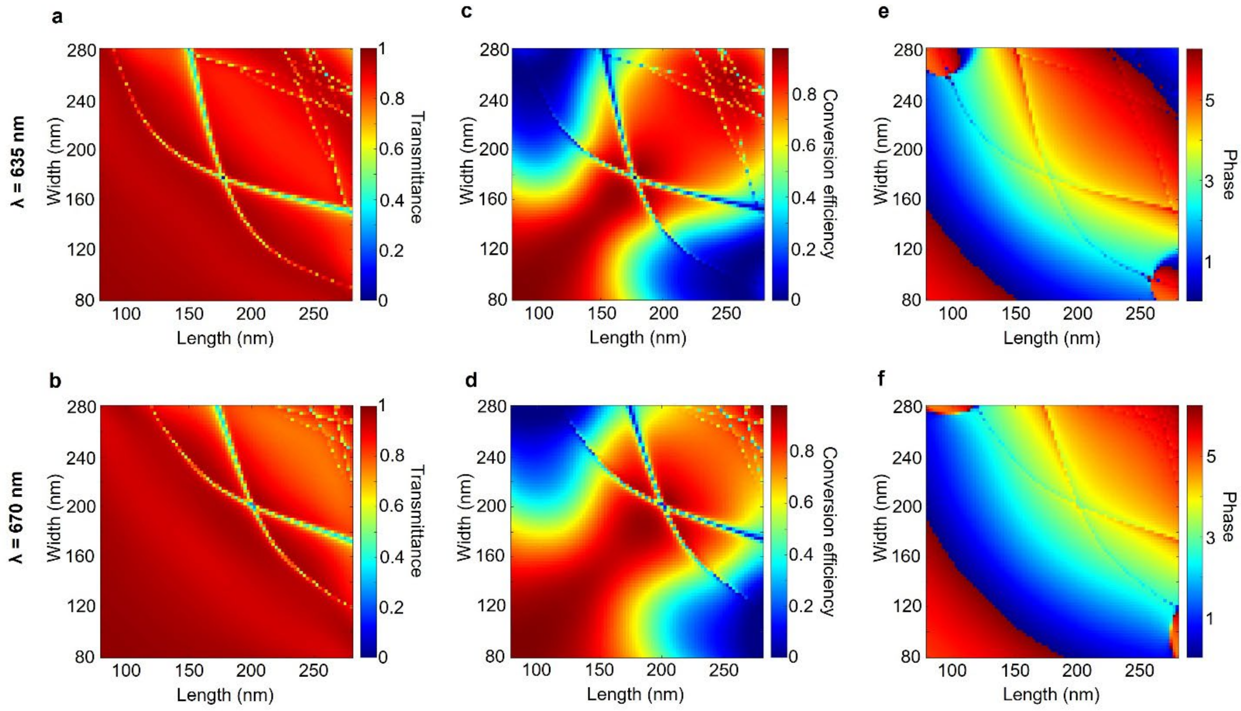

Supplementary Figure 4. (a) Transmittance when sweeping W and L at 635 nm. (b) Transmittance when sweeping W and L at 670-nm incidence. (c) Conversion efficiency when sweeping W and L at 635-nm incidence. (d) Conversion efficiency when sweeping W and L at 670-nm incidence. (e) Phase when sweeping W and L at 670-nm incidence. (f) Phase when sweeping W and L at 670-nm incidence.

Supplementary Note 5. Maps of meta-atom widths and lengths corresponding to matched phase map

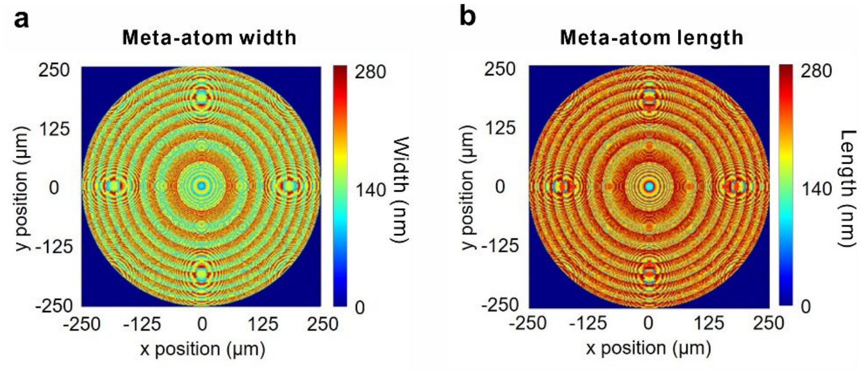

Supplementary Figure 5. (a) Meta-atom width data corresponding to matched phase. (b) Meta-atom length data corresponding to matched phase.

Supplementary Note 6. Efficiency and transmission of matched phase map

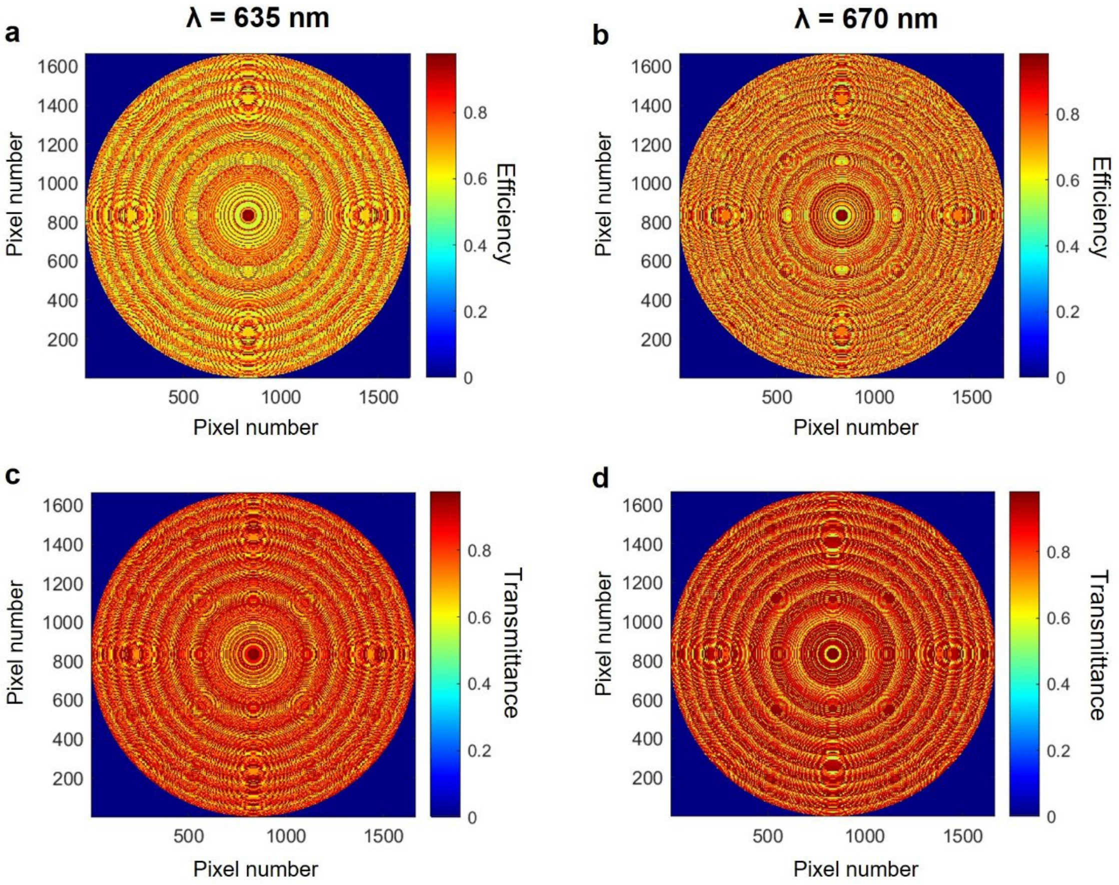

Supplementary Figure 6. (a) Conversion efficiency at 635 nm incidence. (b) Conversion efficiency at 670 nm incidence. (c) Transmittance at 635 nm incidence. (d) Transmittance at 670 nm incidence.

Supplementary Note 7. Comparing conversion efficiencies between co-pol and cross-pol

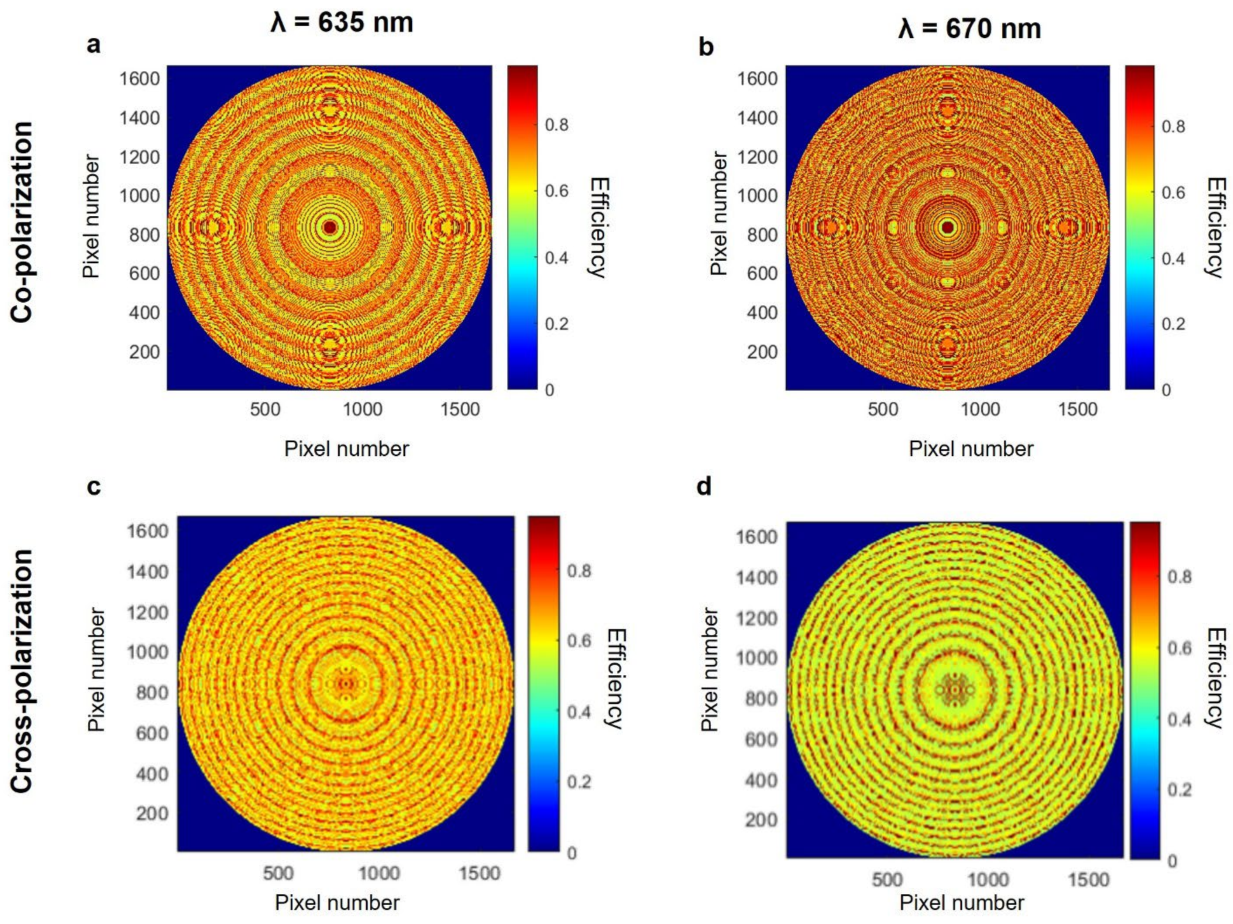

Supplementary Figure 7. (a) Conversion efficiency at 635-nm incidence by using co-polarization term. (b) Conversion efficiency at 670-nm incidence by using co-polarization term. (c) Conversion efficiency at 635-nm incidence by using cross-polarization term. (d) Conversion efficiency at 670-nm incidence by using cross-polarization term. The average conversion efficiency at 635-nm of (c) is 0.6582, and that of (d) is 0.6221.

Supplementary Note 8. Metalens characterization setup

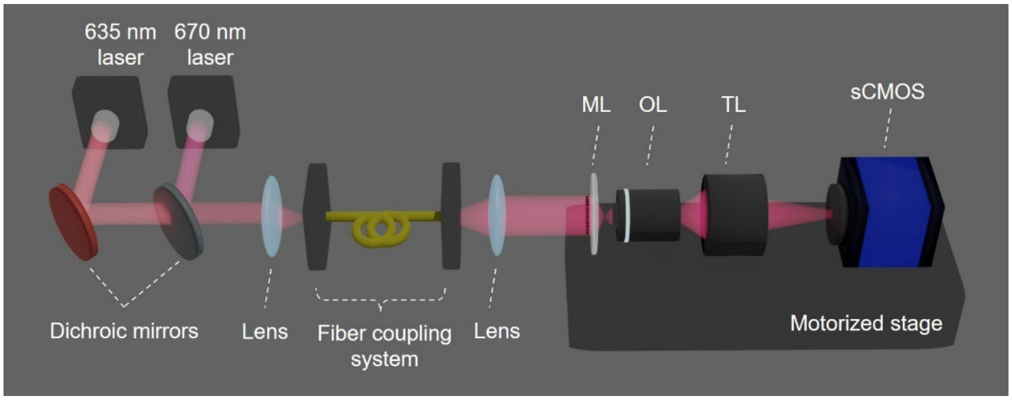

Supplementary Figure 8. Home-built optical transmission microscope for determination of metalens PSF at 635 nm and 670 nm. ML: metalens, OL: objective lens, TL: tube lens.

Supplementary Note 9. Investigation of reasons for enlargement of the focal spot

The observation that the metalens, despite having a specified numerical aperture (NA) of 0.6, exhibits a focused laser spot size significantly larger than $\lambda/2NA$, warrants a comprehensive investigation. Several factors may contribute to this discrepancy, with the primary reasons being phase mismatch and manufacturing imperfections, both of which significantly contribute to the enlargement of the focal spot. The phase mismatch arises due to geometric limitations encountered during the fabrication of nanostructures when accounting for the material's n and k values. To elaborate, our ability to create nanostructures using electron beam lithography (EBL) is bound by the constraints imposed by amorphous silicon (aSi:H), thereby determining the achievable dimensions in terms of length, width, and height. Consequently, practical constraints on the attainable shapes of nanostructures inevitably lead to a disparity between the theoretical phase map and the actual matched phase map. As illustrated in the figure below, a conspicuous disparity exists when comparing the theoretical phase difference map with the post-matching phase difference map.

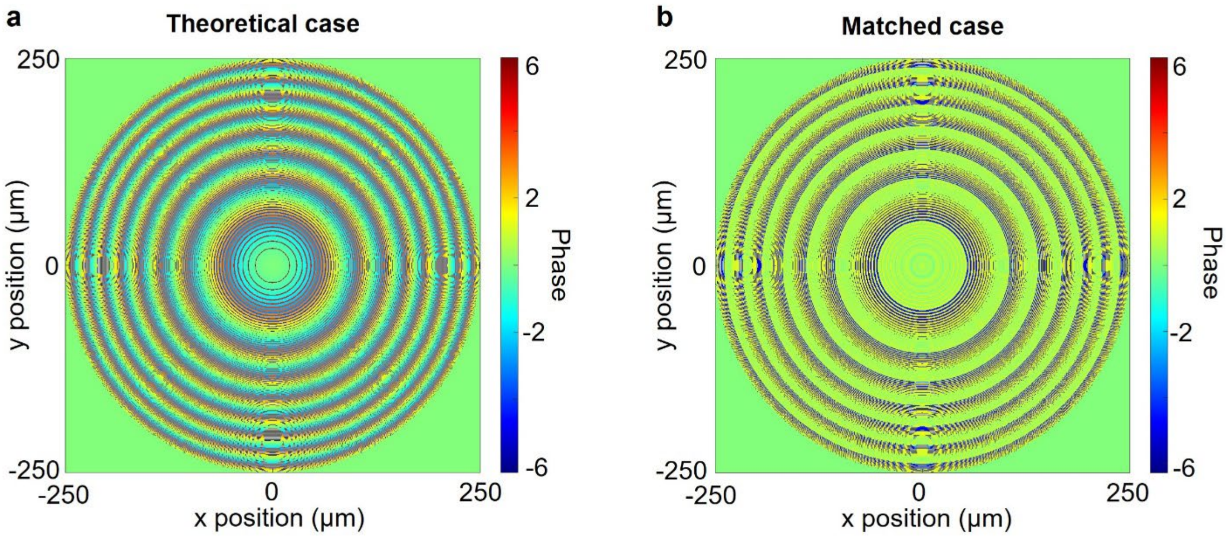

Supplementary Figure 9. (a) Phase difference between 635 nm and 670 nm phase maps in theoretical case. (b) Phase difference between 635 nm and 670 nm phase maps in matched case.

Manufacturing imperfections come from variations in geometry between the target nanostructure and the fabricated counterpart. As depicted in the figure below, while our intended structure is a perfect cuboid, the fabricated structure appears more rounded, with oblique sides deviating from the ideal 90-degree angle due to imperfect etching conditions. This discrepancy induces unintentional phase modulation, significantly influencing the broader full-width-half-maximum observed in our metalens. Additionally, edge effects, stemming from non-ideal behaviors near the lens periphery, and grating effects, especially in nanoscale structures, may affect the focal properties.

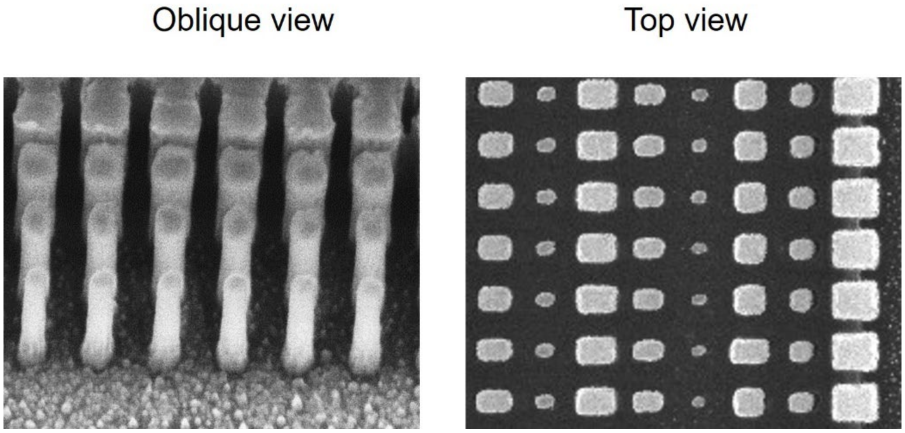

Supplementary Figure 10. The oblique and top view of fabricated nanostructure. The rectangular-shaped meta-atom design is shown in Figure S3.

Supplementary Note 10. Point spread function of theoretically ideal metalens

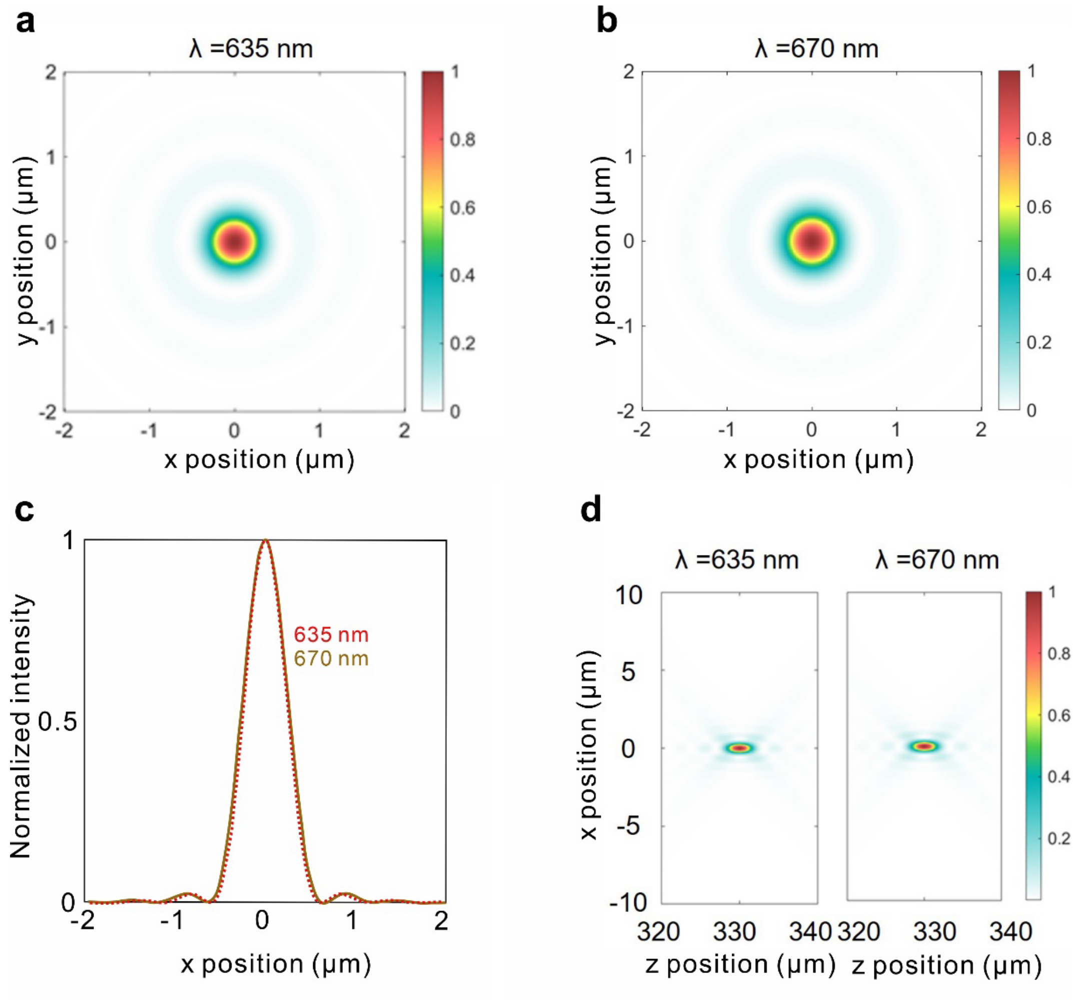

Supplementary Figure 11. (a) 2-dimensional intensity distribution at focal plane at 635-nm incidence. (b) 2-dimensional intensity distribution at focal plane at 670 nm incidence. (c) Line distributions in focal plane center. (d) Scanning results in z-direction.

Supplementary Note 11. Comparison of metalens performance to prior arts

Supplementary Table 1. Chromatic aberration correction, size, NA, and focusing efficiency of the metalenses presented in this work and prior arts.

Operating wavelengths (nm)	Feature	Size (μm)	Effective NA	Focusing efficiency (%)	Reference
635 / 670	Single layer	500	0.6	14.1 / 37.1	This work
470 to 670	Achromatic; single layer	220	0.02	~20	[1]
465 / 548 / 600 / 620	Inverse design, single layer	115	0.3	42.8 / 42.4 /38 / 33.3	[2]
1180 / 1400 / 1680	Vertically stacked multi-layer	120	0.29	34.5 / 30.7 / 51.1	[3]
488 / 532 / 633	Double layer	1000	0.55 / 0.55 / 0.58	12.81 / 13.30 / 42.05	[4]
1200 to 1650 nm	Achromatic; single layer	100	0.24	~35	[5]

Supplementary Note 12. Fluorescence intensity dependence on incident laser power

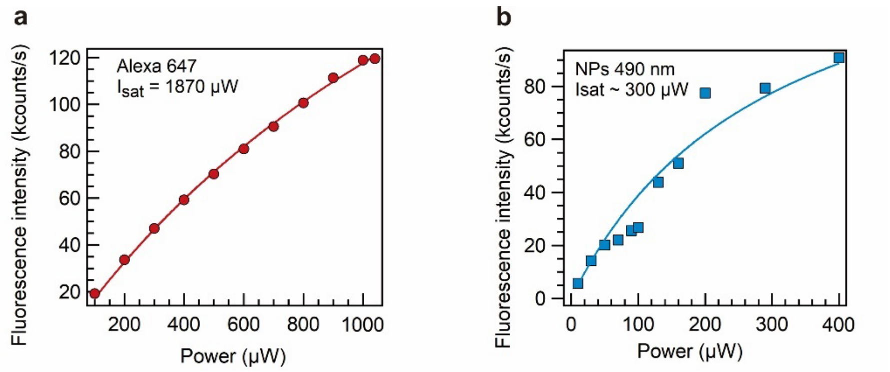

Supplementary Figure 12. (a) Dependence of collected fluorescence intensity of Alexa 647 on laser power sent toward metalens. (b) Dependence of collected fluorescence intensity of nanoparticles (diameter 490 nm) on laser power sent toward metalens. Pulse repetition rate is 40 MHz. Fluorescence intensity is fit by the following equation:[6] $F = \frac{aI}{1+I/I_{sat}}$ with a being product of quantum efficiency of emitter, number of emitters, and collection efficiency of optical system; I is excitation intensity, I_{sat} is saturation intensity.

Supplementary Note 13. Background FCS correlation functions

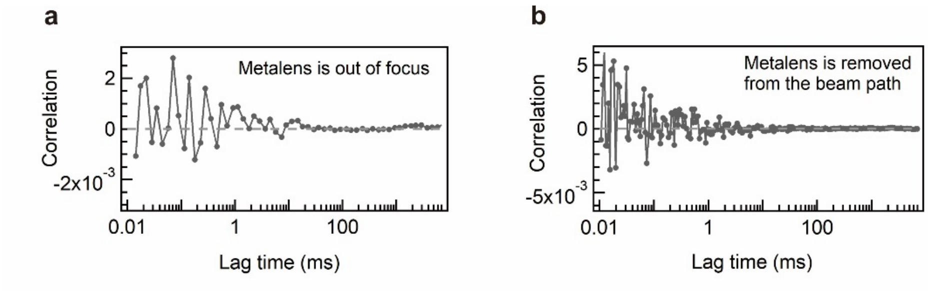

Supplementary Figure 13. (a) FCS correlation function of diffusing Alexa 647 molecules when metalens is out of focus. (b) FCS correlation function of diffusing Alexa 647 molecules when metalens is removed from beam path. Signal acquisition time is identical to FCS measurements shown in Figure 4.

Supplementary Note 14. FCS and TCSPC fit parameters

Supplementary Table 2. Metalens FCS fit parameters of diffusing Alexa 647 molecules/ QDs/ NPs. Dimension of Hz is referred to as fluorescence intensity as counts/s. Parameter κ is set to 10 for all fits.

Fluorescent object	Concentration	F (kHz)	B (kHz)	τ_d (ms)	1/G(0)	N_{mol}	CRM (Hz)
Alexa 647	6 nM	134.6	47.3	3.5	5861	2465	36
	3.5 nM	83.6	34.7	3.5	4197	1435	34
	2 nM	45.1	18.2	2.7	2295	816	33
	1 nM	23	11.9	2.9	1949	445	26
	500 pM	16.2	10.6	3.6	1436	172	33
	100 pM	2.9	1.77	2.7	165	25	45
	50 pM	1.42	0.87	2.2	81	12.2	45
	10 pM	0.72	0.63	2	107	1.7	53
QDs	800 pM	21.2	3.31	20	297	211	85
NPs 110 nm	$C=C_{stock}$	110.4	13.5	147	326	251	386
NPs 490 nm	$C=C_{stock}$	14.6	3	730	2.2	1.3	8550

Supplementary Table 3. Metalens TCSPC histogram fit parameters. Bin time of histograms is 5 ps. FWHM of the instrument response function (IRF) amounts to 160 ps. Fitting undergoes via the exponential reconvolution of IRF. Mono-exponential approximation appropriately fits all the decays except for QDs. Long QD fluorescence decay is well fitted by two exponential components that are frequently used to describe semiconductor quantum dot emission.[7]

Emitting object	τ_1 (ns)	τ_2 (ns)	I_1	I_2	τ_{av} (ns)
Alexa 647 in PBS	0.97		1		0.97
Alexa 647 in glycerol 60 vv%	1.57		1		1.57
QDs	6.5	31.9	0.1	0.9	29.5
NPs 110 nm	4.63		1		4.63
NPs 490 nm	3.47		1		3.47

Supplementary Note 15. Alexa 647 FCS measured by conventional optics

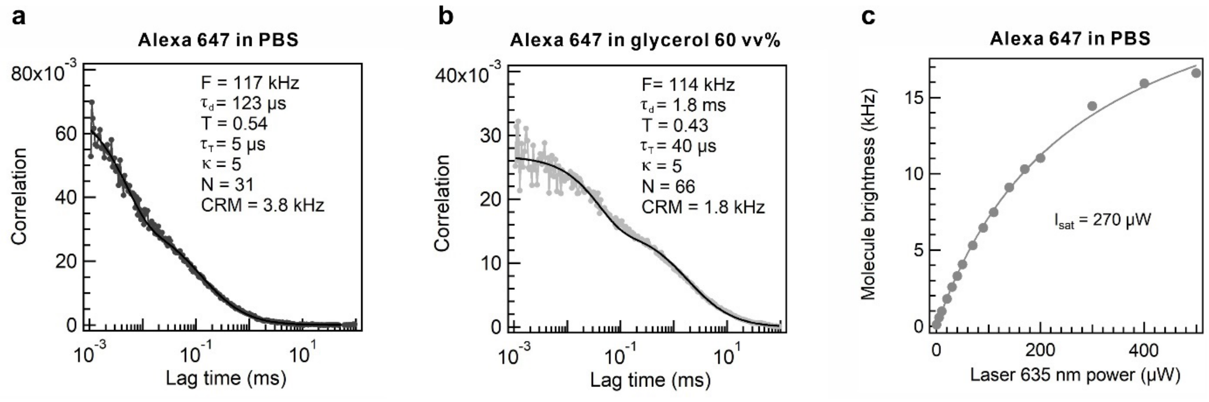

Supplementary Figure 14. (a) FCS correlation function of Alexa 647 (C=10 nM) in phosphate buffer solution (PBS) excited by objective lens NA=1.2, WI. (b) FCS correlation function of Alexa 647 (C=10 nM) in glycerol 60 vv% excited by objective lens NA=1.2. Correlation functions are fitted according to diffusion model with dark state blinking term (Section S6). (c) Single molecule brightness of Alexa 647 in PBS as a function of incident laser power. Saturation intensity amounts to 270 µW at 80 MHz repetition rate. Excitation power for (a) and (b) measurements was set to 50 µW to avoid photobleaching and excessive triplet blinking.

Supplementary Table 4. Metalens size comparison against conventional optics.

Lens type	Numerical aperture	Height (mm)	Width (mm)
Metalens	0.6	$0.5 \cdot 10^{-3}$ (structure)<br>0.5 (substrate)	0.5
Aspheric lenses	0.18	3	7.5
	0.54	9	25
Achromatic objective lenses	0.15	30	25
	0.25	40	25
	0.3	40	25
	0.5	50	25
	0.8	50	25
	0.95	65	33
	1.2	65	34.5

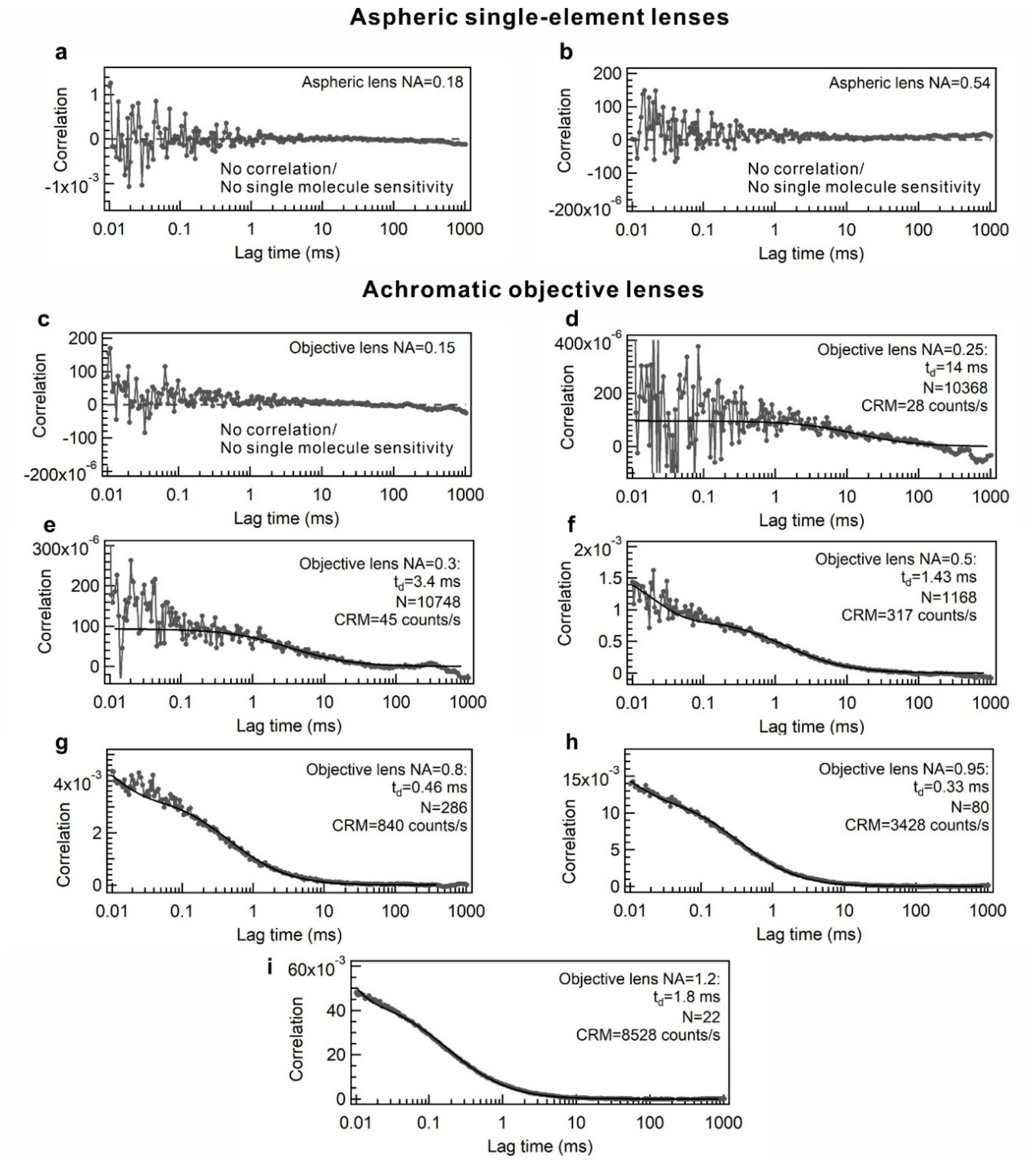

Supplementary Figure 15. (a), (b) FCS correlation functions of diffusing Alexa647 molecules acquired using aspheric lenses of NA=0.18 and NA=0.54, respectively. (c), (d), (e), (f), (g), (h), (i) FCS correlation functions of diffusing Alexa647 molecules acquired using achromatic objective lenses of NA=0.15, NA=0.25, NA=0.3, NA=0.5, NA=0.8, NA=0.95, NA=1.25, respectively. The concentration of Alexa 647 is fixed at 6 nM in these measurements.

Supplementary Note 16. Metalens-recorded fluorescence time traces and FCS of nanoparticles

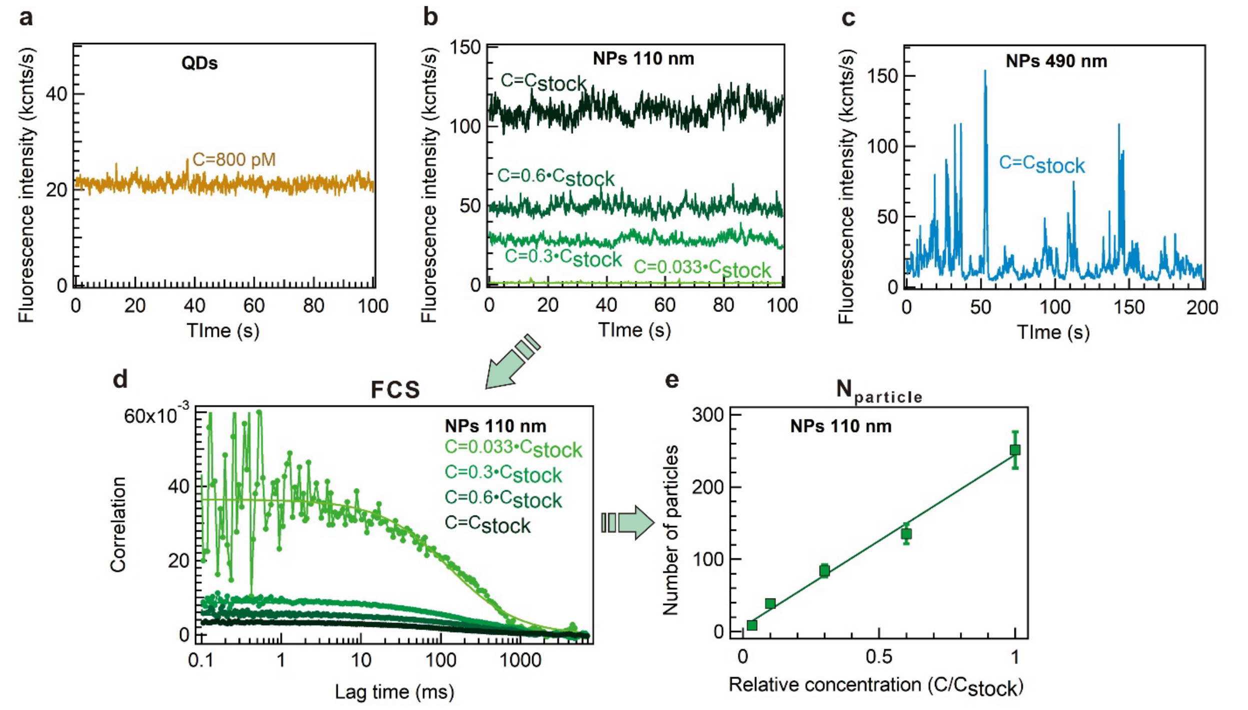

Supplementary Figure 16. (a) Fluorescence intensity time traces of QDs at 800 pM. (b) Fluorescence intensity time traces of NPs 110 nm at various dilutions from stock concentration. (c) Fluorescence intensity time traces of NPs 490 nm at stock concentration. (d) FCS correlation functions of NPs 110 nm retrieved from time traces of (b). (e) Number of nanoparticles determined from the FCS analysis of (b).

Supplementary Note 17. Real-time monitoring of single nanoparticle transits

The experiments of real-time monitoring of single nanoparticle transits were carried out at the excitation power of 100 μW for NPs 110nm and 250 μW for QDs. A stock solution of NPs is dissolved by 200 times and 1000 times to provide 1 particle and 0.2 particles in the detection volume, respectively. QD colloidal solution is diluted to 8 pM and 1 pM to provide 1.3 particles and 0.17 particles in the detection volume, respectively. Owing to low signal, QDs were diluted in glycerol 60 vv% to delay transit time. Bin sizes employed in the time trace analysis equal 200 ms roughly corresponding to diffusion time of nanoparticles.

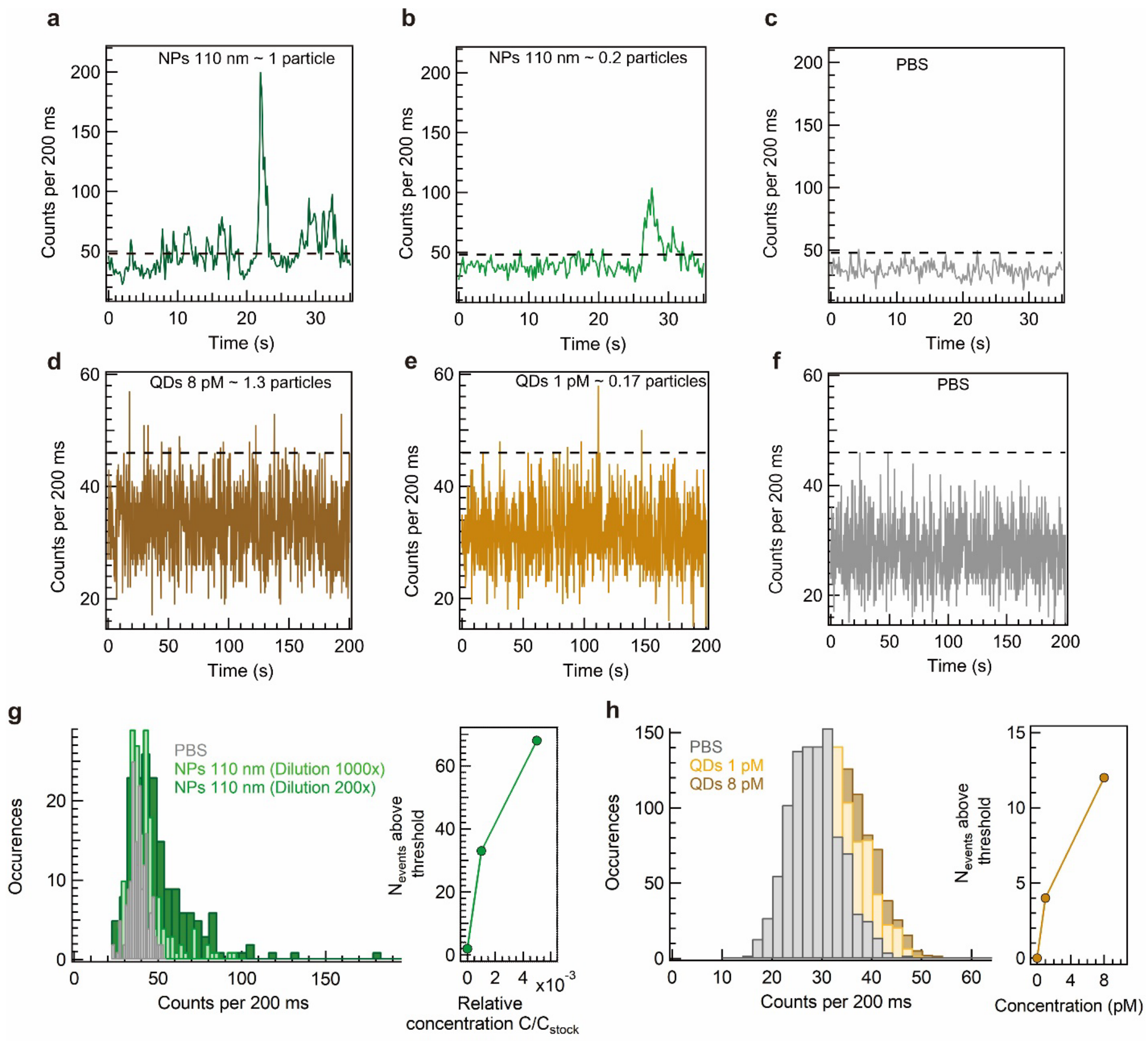

Supplementary Figure 17. Real-time monitoring of NPs 110 nm transits at (a) 1 nanoparticle in detection volume, (b) 0.2 nanoparticles in detection volume, (c) background fluorescence from pure PBS buffer. Real-time monitoring of QDs transits at (d) 1.3 particles, (e) 0.17 particles, (f) pure PBS buffer. (g) Histograms of time traces (a–c) and number of data points above the selected threshold line (dashed line) at two nanoparticle concentrations. (h) Histograms of time traces (d–f) and number of data points above selected threshold line (dashed line) at two QD concentrations.

Supplementary References

1. Chen, W. T. *et al.* A broadband achromatic metalens for focusing and imaging in the visible. *Nature Nanotechnology* **13**, 220–226 (2018).
2. Jiang, Q. *et al.* Multiwavelength Achromatic Metalens in Visible by Inverse Design. *Advanced Optical Materials* **11**, 2300077 (2023).
3. Zhou, Y. *et al.* Multilayer Noninteracting Dielectric Metasurfaces for Multiwavelength Metaoptics. *Nano Lett.* **18**, 7529–7537 (2018).
4. Feng, W. *et al.* RGB achromatic metalens doublet for digital imaging. *Nano Letters* **22**, 3969–3975 (2022).
5. Shrestha, S., Overvig, A. C., Lu, M., Stein, A. & Yu, N. Broadband achromatic dielectric metalenses. *Light: Science & Applications* **7**, 85 (2018).
6. Eggeling, C. *et al.* Analysis of photobleaching in single-molecule multicolor excitation and Förster resonance energy transfer measurements. *The Journal of Physical Chemistry A* **110**, 2979–2995 (2006).
7. Van Driel, A. F. *et al.* Statistical analysis of time-resolved emission from ensembles of semiconductor quantum dots: Interpretation of exponential decay models. *Physical Review B* **75**, 035329 (2007).
